# Haptoglobin Treatment for Aneurysmal Subarachnoid Hemorrhage: Review and Expert Consensus on Clinical Translation

**DOI:** 10.1161/STROKEAHA.123.040205

**Published:** 2023-05-26

**Authors:** Ian Galea, Soham Bandyopadhyay, Diederik Bulters, Rok Humar, Michael Hugelshofer, Dominik J. Schaer

**Affiliations:** Department of Clinical Neurosciences, Clinical & Experimental Sciences, Faculty of Medicine, University of Southampton, Hampshire, United Kingdom (I.G., S.B., D.B.).; Wessex Neurological Centre, University Hospital Southampton NHS Foundation Trust, Southampton, United Kingdom (I.G., S.B., D.B.).; Division of Internal Medicine (R.H., D.J.S.), Universitätsspital and University of Zurich, Switzerland.; Department of Neurosurgery, Clinical Neuroscience Center (M.H.), Universitätsspital and University of Zurich, Switzerland.

**Keywords:** blood, haptoglobins, hemoglobins, subarachnoid hemorrhage, therapeutics

## Abstract

Aneurysmal subarachnoid hemorrhage (aSAH) is a devastating form of stroke frequently affecting young to middle-aged adults, with an unmet need to improve outcome. This special report focusses on the development of intrathecal haptoglobin supplementation as a treatment by reviewing current knowledge and progress, arriving at a Delphi-based global consensus regarding the pathophysiological role of extracellular hemoglobin and research priorities for clinical translation of hemoglobin-scavenging therapeutics. After aneurysmal subarachnoid hemorrhage, erythrocyte lysis generates cell-free hemoglobin in the cerebrospinal fluid, which is a strong determinant of secondary brain injury and long-term clinical outcome. Haptoglobin is the body’s first-line defense against cell-free hemoglobin by binding it irreversibly, preventing translocation of hemoglobin into the brain parenchyma and nitric oxide-sensitive functional compartments of cerebral arteries. In mouse and sheep models, intraventricular administration of haptoglobin reversed hemoglobin-induced clinical, histological, and biochemical features of human aneurysmal subarachnoid hemorrhage. Clinical translation of this strategy imposes unique challenges set by the novel mode of action and the anticipated need for intrathecal drug administration, necessitating early input from stakeholders. Practising clinicians (n=72) and scientific experts (n=28) from 5 continents participated in the Delphi study. Inflammation, microvascular spasm, initial intracranial pressure increase, and disruption of nitric oxide signaling were deemed the most important pathophysiological pathways determining outcome. Cell-free hemoglobin was thought to play an important role mostly in pathways related to iron toxicity, oxidative stress, nitric oxide, and inflammation. While useful, there was consensus that further preclinical work was not a priority, with most believing the field was ready for an early phase trial. The highest research priorities were related to confirming haptoglobin’s anticipated safety, individualized versus standard dosing, timing of treatment, pharmacokinetics, pharmacodynamics, and outcome measure selection. These results highlight the need for early phase trials of intracranial haptoglobin for aneurysmal subarachnoid hemorrhage, and the value of early input from clinical disciplines on a global scale during the early stages of clinical translation.

Aneurysmal subarachnoid hemorrhage (aSAH)—with an incidence of 6.1 per 100 000 person years^1^—carries a higher economic cost than other stroke types^2^ since it has worse outcomes and commonly affects patients of working age. The mainstay of treatment is prevention of rebleeding by securing the culprit aneurysm.^3^ Prophylactic triple-H treatment (hypervolemia, hypertension, and hemodilution) is no longer recommended, and high-quality evidence supporting hemodynamic augmentation methods to treat symptomatic vasospasm is lacking.^3^ Pharmacological treatment with Class I evidence of improving outcome is limited to oral nimodipine.^3^ Despite advances in management such as nimodipine,^4^ specialist care for patients,^5^ and endovascular coiling of ruptured aneurysms,^6^ aSAH still causes significant morbidity in survivors, including disabilities such as cognitive dysfunction,^7^ hearing difficulty,^8^ and fatigue,^9^ which affect quality of life and employment.

There is therefore an unmet need for new treatments for aSAH. While several are under investigation, this Special Report focusses on the development of the hemoglobin-binding protein haptoglobin as a treatment. First, we review hemoglobin’s association with secondary brain injury (SBI) and the evidence supporting the therapeutic potential of intraventricular haptoglobin. Then, we present the results of a Delphi study to determine the global perspective regarding hemoglobin’s role in aSAH and the research priorities for translational development of haptoglobin therapeutics. Translational stroke research has many challenges.^10^ Practising clinicians will ultimately be the deployers of a novel treatment for aSAH, and early input into clinical translational programmes will increase their safety and efficiency while ensuring acceptability by clinical stakeholders.

## Methods

The first part of this paper was synthesized as a narrative review of the topic (by the authors I.G., S.B., D.B., R.H., M.H., and D.J.S.), with the aim of providing the background for the Delphi study (Supplemental Material).

The second part followed a modified Delphi process^11^ with a preprotocolled design (Supplemental Material) to arrive at a consensus regarding: (1) importance of specific pathophysiological pathways leading to SBI post-aSAH; (2) contribution of hemoglobin to the same pathophysiological pathways; (3) research priorities for clinical translation of haptoglobin as a treatment for aSAH.

An online survey platform Welphi was used to implement the Delphi method. Participants’ inclusion criteria were (1) experience with managing aSAH patients from the clinical disciplines of neurosurgery, neurointensive care, neuroradiology, and neurology, (2) scientific expertise (academic or pharmaceutical industry) in the field of hemoglobin/haptoglobin, enabling separate analyses of these 2 groups. Invitations were sent to eligible participants identified through professional networks with a specific effort to ensure global representation. Primary contacts were strongly encouraged to advertise the Delphi in their professional and geographic areas, using a network propagation approach. Four virtual meetings were held, 2 before starting, and 2 during the first round. Otherwise, the study was conducted electronically. Consensus was defined a priori as either consensus in (a priority), consensus out (not a priority) or consensus of equipoise, with scoring criteria for each detailed (Supplemental Material). Ranks and Likert scores were compared with Mann-Whitney *U* test or Wilcoxon tests (alpha=0.05) in STATA/IC version 16.1.

### Root Triggers of Brain Injury

**Figure 1.**
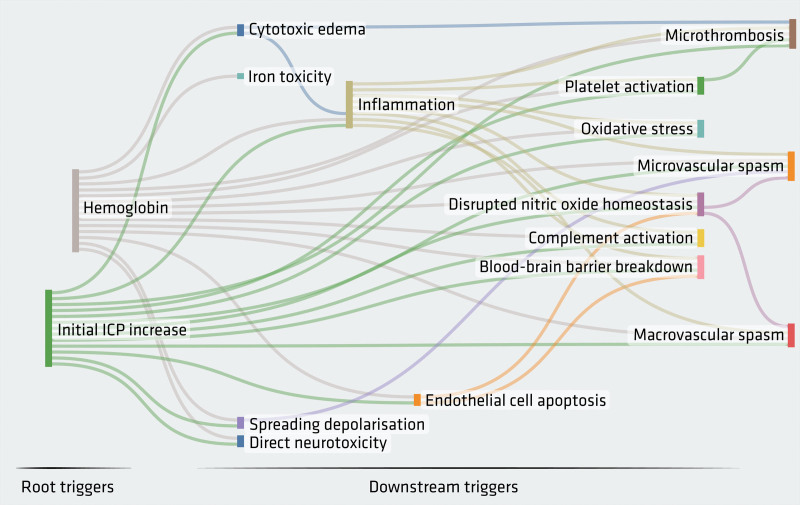
**Root triggers.** Acute intracranial pressure rise and cell-free hemoglobin as root triggers leading to downstream causes of brain injury post-aneurysmal subarachnoid hemorrhage.

The lack of pharmacological treatments with Class I evidence contrasts sharply with the plethora of basic pathophysiological mechanisms described in aSAH^12^ (Figure 1). With many of these, experimental interrogation resulted in short-term improvement in preclinical models, but clinical trials failed to show an effect on long-term outcomes. A picture is emerging that, while these mechanisms are important, it is their combination that determines outcome; therefore, inhibiting 1 pathway is not sufficient. It therefore follows that targeting the root cause of these pathophysiological mechanisms would be a logical approach. Another advantage of targeting an early trigger is that many of the disease processes have the potential to exacerbate each other, leading to amplification of brain injury. Hence the further upstream a therapeutic target is, the less likely such amplification will occur. A root cause is defined by its capacity to lead to downstream events, in the absence of anything upstream to it. Two main root triggers are easily identifiable post-aSAH: (1) intracranial pressure (ICP) rise and (2) blood components. It is important to differentiate between root causes, as defined here, and systemic modifiers of outcome such as arterial stiffness,^13^ viscosity,^14^ hypertension and diabetes,^15,16^ and other comorbidities.^17^

#### ICP Rise

The earliest root trigger is the initial rise in ICP, which immediately causes cerebral ischemia and mechanical tissue damage. This leads to several responses within the first 72 hours such as edema,^18^ spreading depolarization,^19^ inflammation,^20^ platelet activation,^20^ and blood-brain barrier breakdown^.21^ Collectively, the damage induced by these mechanisms has been referred to as early brain injury (EBI). The most important clinical and imaging correlates of EBI are general impairment of neurological status (eg, World Federation of Neurological Societies score) and varying degrees of cerebral edema. EBI is challenging to target therapeutically for several reasons. There is a limited time window before the full pattern of EBI is established, but the root cause of EBI, the ICP rise, is a fait accompli.

The descriptor “delayed” refers to events occurring beyond 72 hours post-aSAH. However, this is an artificial separation; in reality, there is extensive overlap, principally because some processes triggered during EBI such as inflammation continue developing with time. Early events may also lead to other processes, for instance, platelet activation leads to microthrombosis,^22^ and spreading depolarization leads to microvascular ischemia.^23^ The predominant imaging and clinical correlates of delayed brain injury are delayed cerebral ischemia (DCI) causing delayed ischemic neurological deficit and spasm of the large arteries (angiographic vasospasm) and microvasculature (microvascular spasm). These delayed processes may be reversible or lead to SBI.

#### Blood Components

Blood components are the second root trigger and can be mainly divided into plasma proteins and red blood cells. Plasma proteins are damaging to the central nervous system (CNS) at high concentration,^24^ which is possibly why they are excluded by the blood-brain barrier. However, plasma proteins are rapidly cleared from the subarachnoid space post-aSAH,^25^ given that the ventricular system can turn around 500 mL of cerebrospinal fluid (CSF) in 24 hours. Red blood cells are not necessarily damaging in themselves, but over time they lyse releasing their contents of which hemoglobin has been heavily implicated in brain injury. As far back as 1949, different blood components were tested for CNS toxicity after intracisternal injection in dogs, and cell-free hemoglobin was identified as the main culprit.^26^

### Cell-Free Hemoglobin

**Figure 2.**
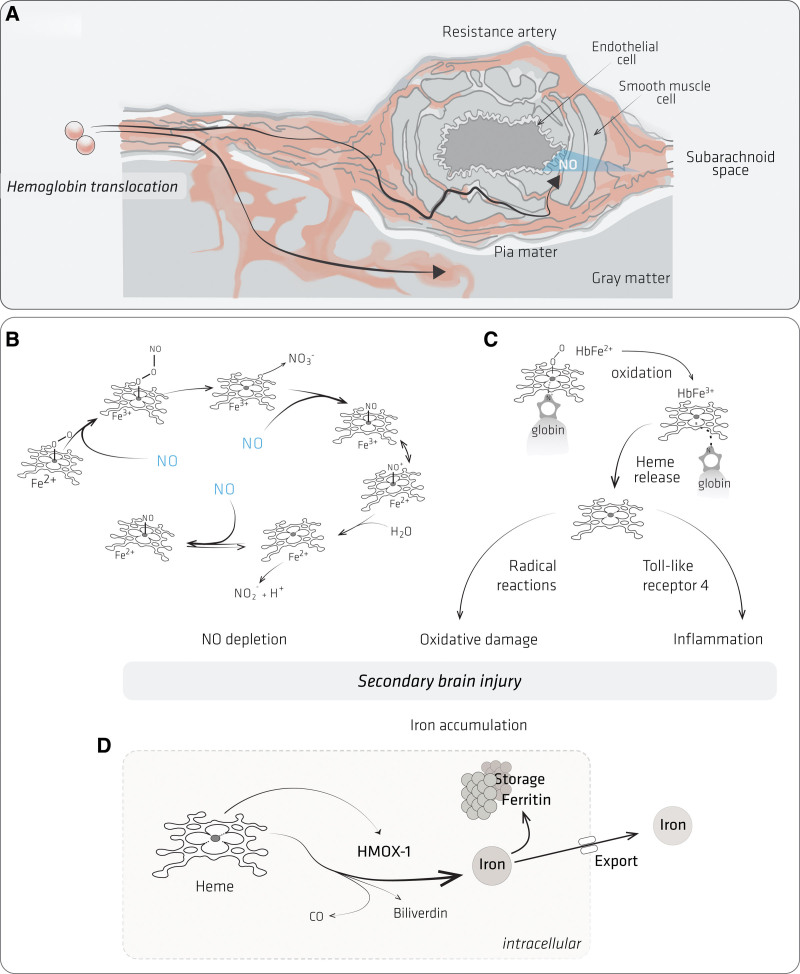
**Mechanisms of Hb toxicity. A**, Hemoglobin dimers in the subarachnoid space can penetrate: (1) the interstitial space between smooth muscle cells of cerebral arteries. Consumption of endothelium-derived nitric oxide causes vasospasm; (2) cortical tissue, promoting oxidative damage and inflammation. **B**, Reactions of NO with Hb across a range of O_2_ liganded [oxy-Hb(Fe^2+^O_2_)] and nonliganded states [deoxy-Hb, Hb(Fe^2+^) and met-Hb [Hb(Fe^3+^)]. The first NO reaction with oxy-Hb produces Hb(Fe^3+^) and nitrate (NO_3_^–^). A second, slower NO consumption step reaction is proposed to involve a series of reaction intermediates, which ultimately react with water and production of nitrite (NO_2_^–^), H^+^, and Hb(Fe^2+^). In a third reaction, NO binds to Hb(Fe^2+^). **C**, Oxidized Hb(Fe^3+^) releases heme, which promotes radical reactions and inflammation via TLR receptor signaling. **D**, In macrophages, heme is metabolized by heme oxygenase (HMOX-1) generating carbon monoxide (CO), bilirubin, and iron. Iron is either exported or stored in ferritin.

In 1976, Miyaoka et al^27^ showed that cell-free hemoglobin was responsible for vasospasm in dogs and humans. More recently, the unique disease-driving function of hemoglobin post-aSAH was reinforced by a mouse model of prolonged intrathecal exposure to hemoglobin, which recapitulated the behavioral, vascular, cellular, and molecular changes seen after human aSAH.^28^ Erythrophagocytes in the blood clot, which ingest decaying red blood cells and detoxify heme^29^ and may exit the CNS via spinal nerve roots,^30^ develop from CNS-resident meningeal and perivascular macrophages^31^ and CSF/blood-derived monocytes,^32^ but microglia may also participate in the process^33^ if there is parenchymal extension of the hemorrhage. However, erythrophagocytosis is unable to clear large blood clots, especially since the CNS is an immune-privileged site with few resident myeloid cells and highly regulated monocyte influx.^34^ Hence, red blood cells start decaying releasing significant quantities of hemoglobin after a lag period of about 3 days.^25,28^ By this time, the blood clot is firmly attached to the brain surface, enveloping cerebral arteries and infiltrating sulci. The close apposition to arteries and cortex facilitates the passage, or delocalization, of small hemoglobin dimers into the cerebral arterial walls and cortical tissue (Figure 2A). Hemoglobin can readily permeate cerebral arterial walls and cortex,^28,35–37^ and there is a clear spatial association between blood clot and subsequent brain iron deposition in the cortex.^37^

Mechanisms driving poor outcome can be mediated via DCI or non-DCI mechanisms, and hemoglobin may play a role in both (Figure 2). Hemoglobin can trigger DCI via nitric oxide consumption (Figure 2A) or spreading depolarization. Oxy-hemoglobin binds to, reacts with, and consumes nitric oxide (Figure 2B), decreasing its bioavailability to smooth muscle cells. This process reverses the basal level of vasodilation mediated by endothelial cell-derived nitric oxide, resulting in vasospasm of both large and small arteries. Since endothelial cell apoptosis occurs post-aSAH,^38^ hemoglobin-induced nitric oxide scavenging occurs on a background of an already dysregulated nitric oxide homeostasis.^39^ The other mechanism is via cortical spreading ischemia, during which spreading depression reverses normal neurovascular coupling from a vasodilatory to a vasoconstrictor response^23^; hemoglobin has been shown to induce spreading depolarization in the presence of a high potassium concentration.^40^ Downstream products of hemoglobin such as bilirubin oxidation products may also cause vasospasm.^41^

Hemoglobin is also a root trigger of direct neurotoxicity and neuroinflammation, independent of DCI. Hemoglobin is directly toxic to neurones in culture^28,42,43^; various mechanisms may account for this direct toxicity, all due to the heme moiety (Figure 2C). While still bound to globin, heme can enter cyclic reactions with hydrogen peroxide or lipid hydroperoxides to generate free radicals, which then self-propagate in the presence of molecular oxygen. Heme-generated free radicals indiscriminately react with lipids, proteins and nucleic acids, resulting in widespread cellular and organ damage.^44^ Molecular signatures of this process, including covalently modified proteins^45^ and oxidized lipids,^46^ have been detected in the CSF post-aSAH. When released from globin, heme is thought to be more toxic than hemoglobin, since it is highly lipophilic easily intercalating into membranes and perturbing cellular function.^47^ Under certain conditions, free heme can also directly induce neuroinflammation via toll-like receptor signaling.^48^ Hemoglobin-treated microglia release soluble factors which induce neuronal necroptosis.^49^ Free labile iron released from heme (Figure 2D) can kill cells by ferroptosis.^50^

While DCI contributes to long-term outcome, a likely dissociation between angiographic vasospasm and outcome is suggested by the nimodipine and clazosentan clinical trials. Nimodipine improved clinical outcome despite having no effect on angiographic vasospasm,^4^ and clazosentan did not improve clinical outcome despite reducing vasospasm.^51^ It is therefore possible that pathophysiological mechanisms, not captured by routine clinical imaging, have a larger effect on clinical outcome, and are important therapeutic targets, especially if several of them can be addressed simultaneously. In this respect, hemoglobin seems to be a master orchestrator of brain injury, playing a role in vasospasm and ischemia as well as direct neurotoxicity, inflammation, and other processes set in motion during EBI (Figure 1).

Recent data support the role of hemoglobin post-aSAH in humans. In 42 patients undergoing serial CSF sampling, there was a significant temporal association between peak concentration of CSF hemoglobin (CSF-Hb) and subsequent CSF neurofilament light chain, a marker of neuronal damage and a strong independent predictor of long-term outcome post-aSAH.^52^ In another cohort of 47 patients, CSF-Hb was associated with the composite outcome of delayed ischemic neurological deficit, radiological evidence of infarction and angiographic vasospasm.^25^ A CSF-Hb concentration of 7.1 µmol/L yielded the best combination of specificity and sensitivity for developing SBI and this level of CSF-Hb was the same as the critical inflection points associated with pronounced hemoglobin-induced vasoconstriction and lipid peroxidation in ex vivo experiments. An ongoing study aims to prospectively validate and conclusively determine the targetable concentration of CSF-Hb post-aSAH.^53^

### Haptoglobin in the CNS

**Figure 3.**
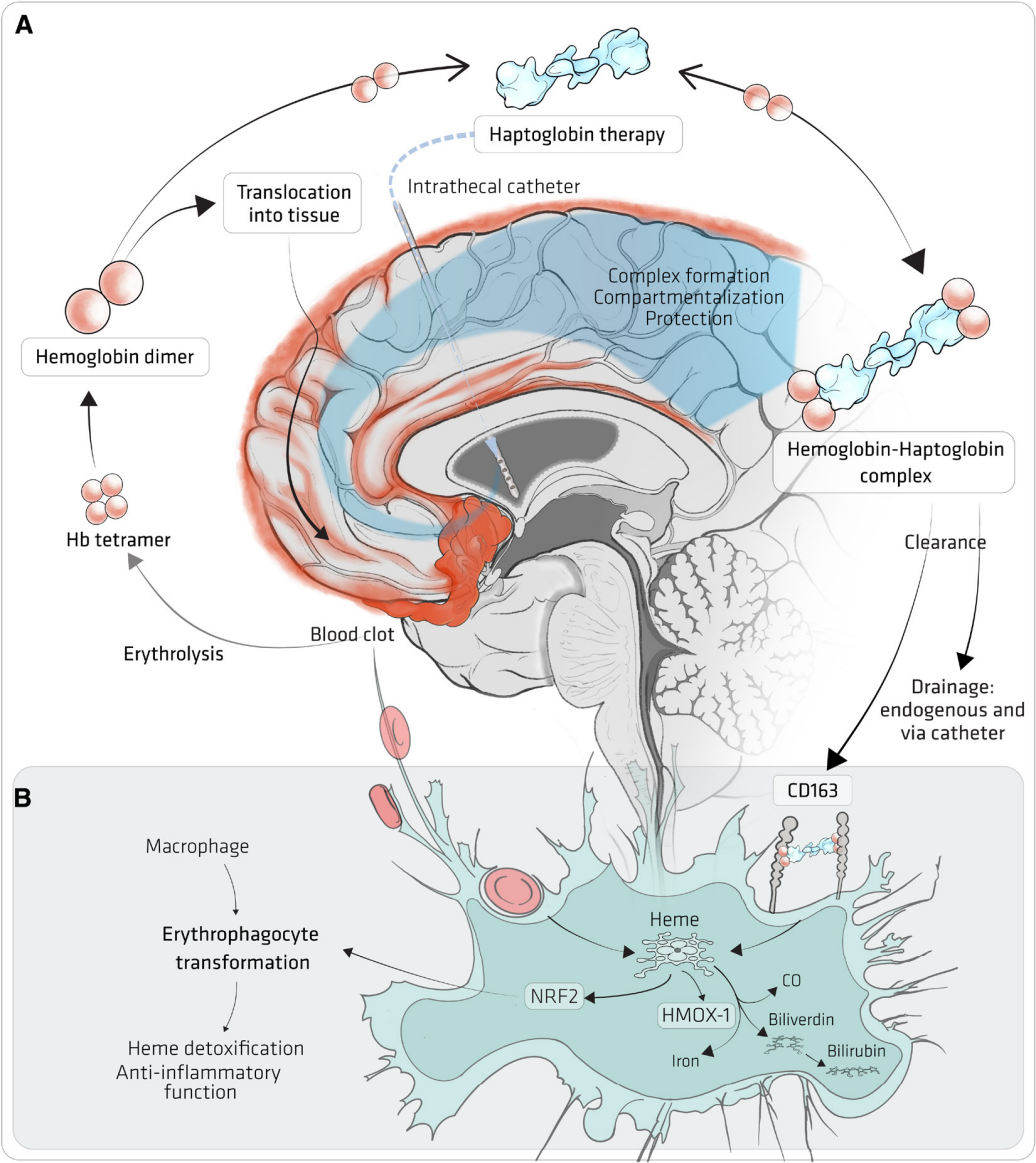
**Haptoglobin treatment. A**, SAH after aneurysmal rupture forms a blood clot. Erythrolysis releases hemoglobin tetramers, which dissociate into dimers in CSF (red in the online version). Small Hb dimers penetrate the brain parenchyma and NO-sensitive arterial compartments to cause secondary brain injury. Therapeutic haptoglobin (blue in the online version) administered via an intrathecal catheter distributes throughout the cerebrospinal fluid (CSF) compartment and binds free hemoglobin. The large hemoglobin-haptoglobin complex remains confined outside the parenchyma and vulnerable arterial compartments, thereby protecting from hemoglobin-induced damage. The hemoglobin-haptoglobin complex is cleared by physiological drainage pathways and drained through intraventricular and/or lumbar catheters (Figure S1). **B**, The role of macrophages in erythrophagocytosis and hemoglobin-haptoglobin complex clearance. Following degradation, heme is metabolized to bilirubin, carbon monoxide, and iron through heme-mediated induction of HMOX-1 (heme-oxygenase 1). Heme-induced activation of NRF2 (nuclear factor erythroid 2-related factor 2) signaling induces an anti-inflammatory macrophage phenotype (ie, erythrophagocyte).

Nature has evolved a multi-tier system to protect against the toxicity of extracellular hemoglobin.^54^ The first line of defense is provided by haptoglobin binding, which stabilizes hemoglobin structure, prevents heme-release, and neutralizes hemoglobin’s oxidative potential.^55^ Most importantly, the large size of the complex inhibits permeation of hemoglobin into tissue (Figure 3). The haptoglobin (*HP*) locus is polymorphic with 2 main variants: *HP1* and *HP2*. These alleles result in protein monomer units with 1 or 2 cysteine residues, respectively, such that *HP1* homozygotes produce dimers, *HP2* homozygotes produce tetramers and higher order polymers up to 20-mers, and heterozygotes (*HP2-1*) produce all varieties. In the extracellular space, the quaternary structure equilibrium of hemoglobin shifts from tetramers to dimers, which can be bound at a ratio of 1 hemoglobin dimer per haptoglobin monomer subunit. Haptoglobin-hemoglobin binding is essentially irreversible, and complexes are cleared by the scavenger receptor CD163,^56^ expressed mostly by macrophages (Figure 3B). If the haptoglobin-CD163 system fails, heme is released in the extracellular space, and the second line of defense is hemopexin, which binds heme and targets it to clearance by CD91.^57^

The highly specialized nature of the CNS has the consequence of reducing its capacity to deal with extracellular hemoglobin in several ways.^54^ First, while haptoglobin is an abundant protein in plasma, it is present at a very low level in the CNS^58^; the blood-brain barrier keeps protein levels low in the brain, and the high molecular weight of haptoglobin polymers restricts their entry into the CNS. Second, CD163-binding sites in the brain are sparse compared with the rest of the body, including post-aSAH; this is mostly a consequence of the brain’s immune privilege with low numbers of CD163-positive macrophages, but also because of enzymatic CD163 shedding by ADAM17 post-aSAH.^58^ Finally, solute drainage is slow from the brain (Figure S1), due to a low interstitial fluid turnover (0.1% per minute)^59^ and low CSF turnover (0.3% per minute) compared with interstitial fluid turnover in some other organs (up to 2.5% per min in kidney).^60^ As a result of these 3 features, most CSF-Hb post-aSAH is not bound by haptoglobin,^58^ and whether bound to haptoglobin or not, CSF-Hb concentrations are sustained for a long time by slow clearance and continuous release from the blood clot.

The different haptoglobin phenotypes bind hemoglobin with identical affinities but may have functional differences related to hemoglobin-binding capacity, protection against hemoglobin-induced neurotoxicity, binding of complexes to CD163, subsequent endocytosis, and the ability to generate anti-inflammatory responses. A lower hemoglobin-haptoglobin complex concentration was found in the CSF of *HP2-2* individuals post-aSAH,^61^ but proinflammatory cytokine levels were higher with this *HP* genotype.^62^ These opposing effects may explain why a careful preprotocolled individual patient level data meta-analyses, the largest to date, did not identify significant differences in vasospasm, DCI, or long-term outcome post-aSAH between *HP* genotypes.^63^ A subsequent study showed an interaction between *HP* genotype and Fisher grade, with a protective effect of *HP2-2* in high Fisher grade patients (larger blood clot) when followed up for longer than 2 years but not less.^61^ In summary, the effect of *HP* genotype on outcome is not convincing, possibly because there is very little haptoglobin in the CNS to make a substantial difference to outcome.

### Haptoglobin as a Treatment for aSAH: Proof of Principle Studies

Several in vitro studies have shown that haptoglobin prevents hemoglobin-induced neurotoxicity^28,42,43^ and vasospasm.^27^ A beneficial effect of haptoglobin during intrathecal hemoglobin exposure was first demonstrated in dogs.^27^ In a mouse model, intraventricular haptoglobin administration prevented or attenuated hemoglobin-induced behavioral deficits, small-vessel vasospasm and astrocytic, microglial, and synaptic changes.^28^ In a sheep model, intraventricular haptoglobin administration inhibited hemoglobin-induced angiographic vasospasm by preserving vascular NO signaling.^35^ In both mouse and sheep models, haptoglobin attenuated hemoglobin-induced pathology by preventing delocalization of hemoglobin from CSF into the brain parenchyma. In sheep, haptoglobin also prevented CSF-Hb from reaching the NO-sensitive compartment of larger cerebral arteries. This was consistent with the activity of haptoglobin to restore NO-dependent vasodilatory function in porcine basilar arteries that were immersed in hemoglobin-rich CSF from aSAH patients.^25,35^ One report suggested that haptoglobin increases the vulnerability of CD163-expressing neurons to hemoglobin in vitro by an iron-dependent mechanism.^64^ However, CD163 is not expressed by healthy human neurones and neuronal CD163 expression post-aSAH is exceptionally rare, and when present is limited to areas with extensive neuropil destruction.^37^ Indeed, prolonged intraventricular administration of hemoglobin-haptoglobin complexes to mice in vivo did not result in neuronal loss, synaptic damage, or behavioral change, and iron deposition decreased.^28^

Haptoglobin can be extracted from pooled plasma to a high level of purity^65^ or manufactured recombinantly.^66^ Intravenous administration of haptoglobin has been approved, marketed and used clinically in Japan since 1984, for systemic hemolysis accompanying several systemic conditions.^67^

One 1979 Japanese study tested intracranial administration of a predominantly Hp2-2 plasma-derived preparation in 27 aSAH patients,^36^ providing evidence for haptoglobin’s safety via this delivery route. Haptoglobin was applied to the neck of the aneurysm intraoperatively, after clipping. This was followed by a 2-day infusion via a catheter in the basal cisterns. Doses were not specified. A comparison between arterial diameter on preoperative and postoperative angiograms was used to assess response. Patients were divided into 4 groups depending on the clinical scenario. Of the 5 patients who were operated within the first 48 hours, and therefore received haptoglobin early, only 1 experienced benefit. The other patients, who were operated on later, were divided into 3 groups, depending on whether vasospasm was progressing (n=7), improving (n=10), or unchanged (n=5) at the time of initiation of haptoglobin treatment. The improvement noted in these groups was 5/7 (71%), 9/10 (90%), and 1/5 (20%), respectively. Notably, the latter group of 5 patients were operated on between 7 and 20 days post-aSAH, so haptoglobin was administered too late to prevent the main peak in CSF-Hb. Overall, this preliminary study suggests that optimal timing and dosing of haptoglobin treatment may be crucial to achieve maximal beneficial effects.

### Haptoglobin as a Treatment for aSAH: Practical Considerations

Post-aSAH, the optimum time to start haptoglobin treatment is likely just before CSF-Hb starts rising, before the third post-ictal day. Having up to 3 days to start treatment provides sufficient time for patient transfer to a tertiary center, appropriate preassessment and treatment initiation. Early treatment may be beneficial to build up a higher concentration of haptoglobin and reach areas where CSF circulation has been disrupted by the clot itself. Intrathecal administration would be essential since haptoglobin is a large molecule and has low blood-brain barrier permeability. The ventricular route is theoretically preferable over lumbar administration, to achieve maximum bioavailability around the cortex and within brain sulci. This is supported by data on intraventricular administration of other molecules like cerliponase alfa, an enzyme replacement treatment for CLN2-type Batten’s disease, which in nonhuman primate studies resulted in better brain penetration than lumbar administration.^68^ However, lumbar administration remains less invasive and carries lower risks, and it remains possible that lower but still sufficient levels can be achieved via this route. Alternative modes of administration with practical limitations include intravenous with ultrasound blood-brain barrier opening, direct instillation in the surgical field, and infusion via a subarachnoid catheter. Calculations show that a total haptoglobin dose of 3.5 grams would be needed to bind all hemoglobin released from an average hemorrhage volume of 20 milliliters.^69^ The dose could potentially be tailored to the individual patient, based on blood clot volume estimated from the CT scan upon hospital admission, or individual daily doses could be titrated to the amount of unbound hemoglobin in the CSF effluent during ventricular drainage. CSF-Hb levels post-aSAH are highly variable and may remain high for up to 2 weeks in severe cases.^25,28^ A study of intraventricular hemorrhage suggested that complete clot resorption takes 2 weeks,^70^ but clot resorption is possibly accelerated with haptoglobin treatment. Altogether, it appears that there is scope for personalization of dose and duration of treatment.

With intraventricular administration, CSF haptoglobin levels will be higher, and functional differences between haptoglobin types may become apparent. One unequivocal difference between haptoglobin types is molecular weight, which increases by hemoglobin binding. In HP1-1 individuals, the haptoglobin dimer-hemoglobin complex molecular weight is 153 kDa (89+[32]_2_), while in HP2-2 individuals, the molecular weight of the complex varies between 327 kDa (199+[32]_4_) for the tetramer to >1000 kDa for higher-order multimers.^71^ Movement of interstitial solutes in the brain is dependent on their molecular weight,^72–74^ and it may be argued that haptoglobin dimer in the interstitium would be better at clearing hemoglobin out of the brain. However, the rationale for intraventricular administration of haptoglobin is the size-dependent sequestration of CSF-Hb within the subarachnoid space, before it delocalizes into the brain parenchyma or cerebral artery walls. A more important consideration in aSAH may be the penetration of the blood clot, especially at the interface with the cortex. Therefore, the ideal haptoglobin formulation is one with a molecular size large enough to prevent tissue translocation of CSF-Hb, but small and homogeneous to optimize viscosity and drug distribution within the clot and subarachnoid space.

There is a paucity of population-based pharmacokinetic studies of intraventricular administration of soluble therapeutics in humans or gyrencephalic animals, and no study molecules as large as haptoglobin. The most relevant data is that of cerliponase alfa, which has a molecular weight of 60kDa.^75^ Allometric scaling of dose and pharmacokinetic parameters was used to plan Phase I trial of cerliponase alfa, and retrospective analysis showed near-perfect prediction.^17^ However alterations in the CSF circulation were observed in a nonhuman primate aSAH model, so that penetration of CSF into the brain parenchyma was reduced.^76^ Also, population-based pharmacokinetic modeling of the gadolinium-based MRI contrast agent gadobutrol in humans shows marked differences between noncommunicating hydrocephalus (common post-aSAH) and controls.^77^ Hence pharmacokinetics may differ considerably between the healthy state and post-aSAH, especially of a high molecular weight substance. This may necessitate the need for empirical measurement in the specific case of aSAH, starting with doses calculated from physiologically-based pharmacokinetic models, followed by a predict, learn, confirm approach.^78^

Theoretical adverse events associated with haptoglobin treatment include immunogenicity, hydrocephalus, and increased brain iron deposition. While their risk may be mitigated, a Phase I trial is needed to assess safety. Being an endogenous protein already present in the brain somewhat de-risks immunogenicity. Although discordance between the phenotype of haptoglobin administered and the patient’s *HP* genotype may occur, this is not of concern in transfusion medicine. A higher CSF protein content during treatment with haptoglobin may theoretically predispose to hydrocephalus, although higher protein concentrations than those anticipated are observed in other neurological conditions without this complication. Any risks associated with a high CSF protein could be reduced by a personalized treat-to-target approach and stopping treatment before drain removal to allow a reduction in protein level. Brain iron deposition secondary to hemoglobin-haptoglobin complex metabolism by macrophages is another possibility, which may be mitigated by regular unclamping of the external ventricular drain and placement of a lumbar drain to encourage craniocaudal flow and external drainage of complexes.

### Toward Clinical Translation: Delphi Consensus

**Figure 4.**
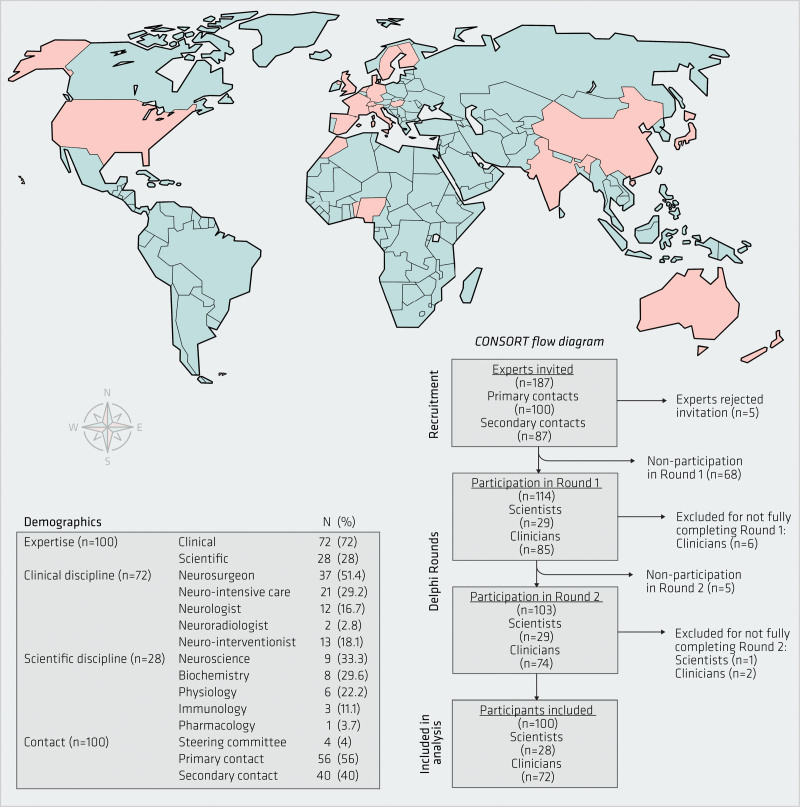
**Delphi study participants.** Geographic distribution and demographics. Please note that clinical disciplines add up to more than 100% since some had dual expertise.

**Figure 5.**
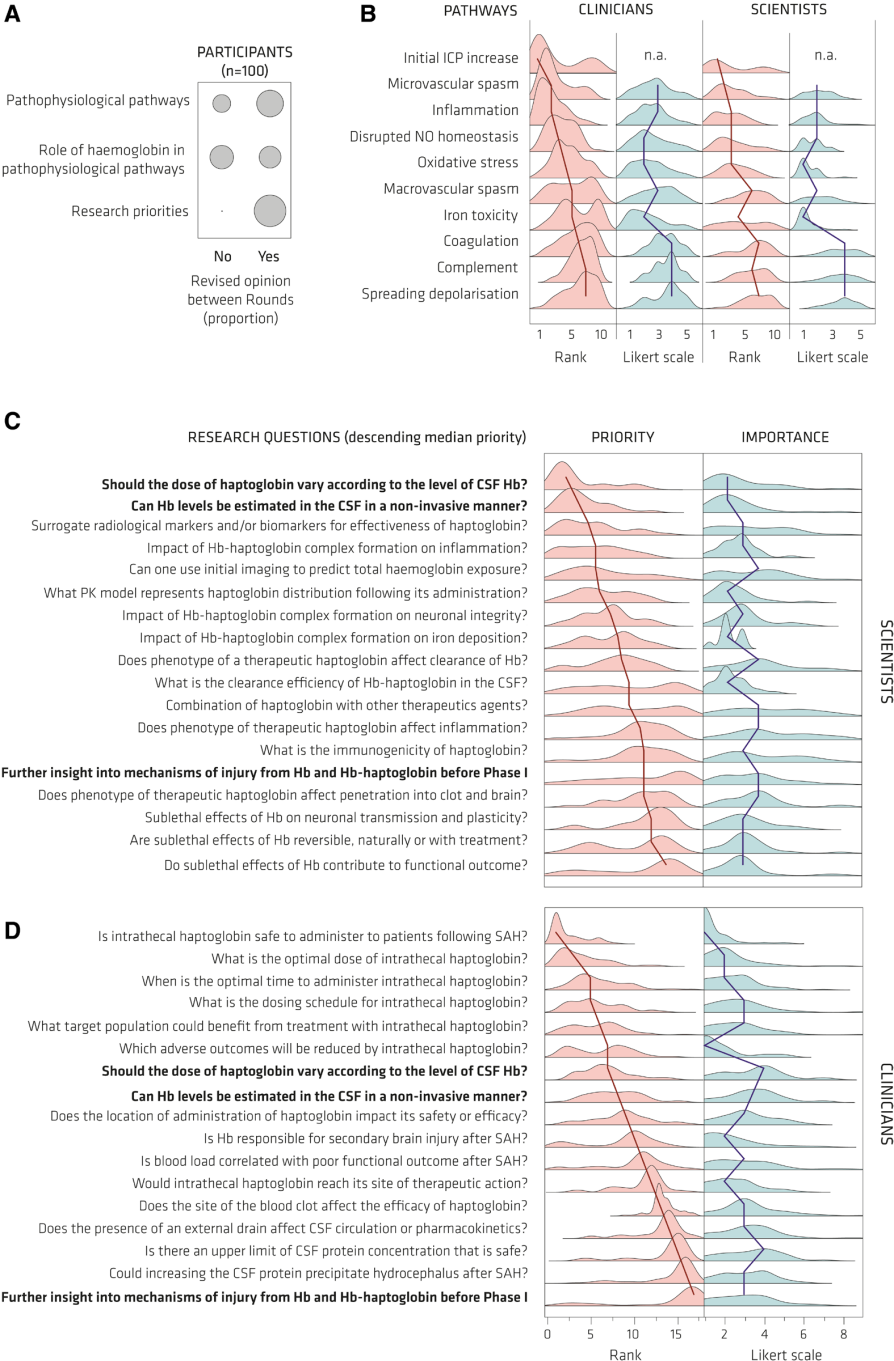
**Delphi study results. A**, Change in responses between Delphi rounds (***P<0.001, Wilcoxon). **B**, Clinicians and scientists were asked to rank 10 pathophysiological pathways according to their role in secondary brain injury (SBI) and estimate the role of hemoglobin for each pathway using a Likert scale with a scoring range from 1 (extremely important) to 5 (not sure). The plots show the kernel density estimation of participants’ responses. The solid line connects the group medians. **C** and **D**, Scientists (**C**) and clinicians (**D**) ranked potential research questions according to their priority and estimated the importance of each research question with a Likert system with a scoring range from 1 (extremely important) to 9 (extremely unimportant). The plots show the kernel density estimation of participants’ responses. The solid line connects the group medians. CSF indicates cerebrospinal fluid.

To achieve a consensus regarding clinical translation, a Delphi study was conducted and completed by 100 individuals (Figure 4). More were clinicians (n=72/100, 72%) than scientists. Participants were from 20 countries across 5 continents. A substantial proportion (n=40, 40%) were not primary contacts, confirming the network propagation technique for identifying participants worked well. Core principles of the Delphi process were upheld. All participants left at least 1 comment in Round 1, and 66% left further comments in Round 2. Five new points were suggested between the 2 Rounds (Figures S2A, S2B, S3A, and S3B), and participants revised their opinion between Rounds (Figure 5A).

All participants agreed there is an ongoing need for novel therapies to prevent or treat SBI in patients with aSAH, and 97% (n=97) agreed that targeting specific pathophysiological pathways is a promising strategy for novel therapies in aSAH patients.

#### The Role of Specific Pathophysiological Pathways in SBI

In this ranking exercise (Figure 5B), among both clinicians and scientists, 18% commented on the difficulty of ranking, in the absence of evidence comparing pathway contribution. However, consensus in was achieved for inflammation (n=85, 85%) and microvascular spasm (n=77, 77%) as key contributors to SBI. A consensus of equipoise was reached for all remaining pathways. The most important pathways within this latter group were thought to be initial ICP increase (n=68, 68%) and disrupted nitric oxide homeostasis (n=66, 66%). When considered as 2 groups, clinicians and scientists mostly agreed on the relative importance of the different pathways, but they differed significantly when it came to the importance of hemoglobin’s role in most of these pathways.

#### The Contribution of Hemoglobin to Specific Pathophysiological Pathways Post-aSAH

Participants considered the importance of hemoglobin for each pathophysiological pathway using a Likert scale (Figure 5B). Among all participants, consensus in was reached for cell-free hemoglobin’s role in iron toxicity (n=73, 73%), with equipoise on all other pathways. Although not reaching consensus among all 100 participants, hemoglobin was also believed to play an important role in oxidative stress (n=67, 67%), disrupted nitric oxide homeostasis (n=65, 65%), and inflammation (n=60, 60%). There was a deviation in viewpoints between clinicians and scientists. Considering clinicians alone, there was equipoise on all pathways. Among scientists, consensus in was reached for iron toxicity (n=26/28, 92.9%), oxidative stress (n=27/28, 96.4%), disrupted nitric oxide homeostasis (n=24/28, 85.7%), and inflammation (n=24/28, 85.7%), with equipoise on all other pathways. This likely reflects the scientists’ more unified perception of mechanistic literature on hemoglobin toxicity.

#### Research Priorities for Clinical Translation of Haptoglobin as a Treatment for aSAH

Finally, participants’ expert opinions were sought regarding research priorities for translating haptoglobin from bench science to a bedside treatment. Scientists (Figure 5C) and clinicians (Figure 5D) were presented with 2 largely different sets of research questions. All 32 points discussed were thought to be important as judged by Likert scoring (Figures 5C and 5D; Figures S2A and S3A), but it was clear that not all were equally important (Figures 5C and 5D; Figures S2B and S3B). Consensus in was reached for 4 research priorities, and 6 research priorities met the criteria for consensus out (Table). A higher percentage of consensus in or consensus out for research priorities was reached among clinicians (n=7/17, 41.2%) compared with scientists (n=3/18, 16.7%).

**Table.**
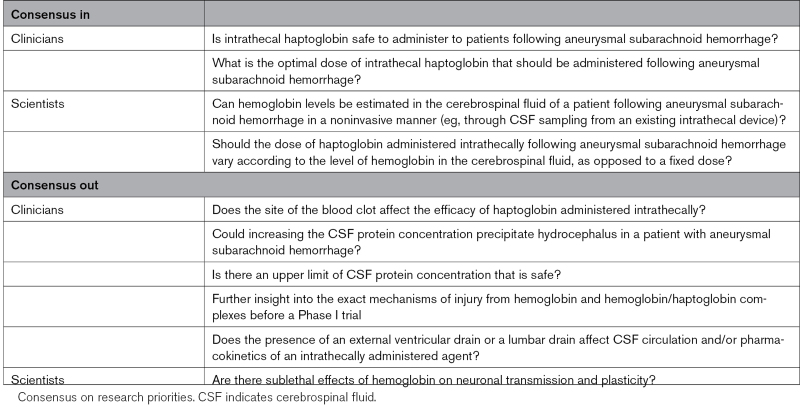
Delphi Study Results

Among the 32 research topics, 3 were considered by both clinicians and scientists: (1) individualized dosing of haptoglobin; (2) estimation of hemoglobin exposure in a noninvasive manner; and (3) the need for further preclinical work before a Phase I trial. There were significant differences between clinicians and scientists (Figures 5C and 5D). Clinicians were more wary of an individualized approach, citing the practical issues for widespread clinical deployment and questioning the validity of any measure of hemoglobin exposure. Scientists acknowledged these difficulties but felt that these research areas were still worth prioritization to better match haptoglobin dose to cell-free hemoglobin concentrations in CSF and minimize any potential adverse events. It was highlighted that proxy measures could be evaluated to calculate hemoglobin exposure, such as blood clot volume on imaging, which might be a compromise that is easier to implement. In the relative ranking of priorities, both expert groups assigned low priority to research focusing on further insight into the exact mechanisms of injury from hemoglobin and hemoglobin-haptoglobin complexes before Phase 1 trial. This may reflect a high confidence in the underlying pathophysiological concepts and a general awareness of limitations of preclinical research predicting success of novel therapeutics.

### Conclusions

This review and Delphi study provide the evidence base for an unmet need for new therapies for aSAH. Research priorities related to 1 such treatment (haptoglobin) corroborated gaps in the existing literature. Some in this Delphi study (n=12/100, 12%) would already advocate for intrathecal haptoglobin as a treatment post-aSAH. This increases to 74/100 (74%) if clinical trial evidence supports its efficacy. All participating clinicians, except for one, would participate in such a clinical trial.

Interpretation needs to be tempered by this study’s limitations. It is possible that these results are not representative of the average clinician looking post-aSAH patients, since sampling of the clinicians was initiated by the authors inviting their contacts. To minimize this bias, a network propagation technique was used and 40% were not primary contacts. Efforts were made to be as geographically inclusive as possible but there is still under-representation from under-resourced countries. Though sample size in Delphi studies is usually driven by practical issues,^11^ n=100 may still be considered to be small in the global context.

This Delphi study demonstrated important differences in perspective between clinicians and scientists regarding pathophysiology and design of a Phase I/IIa trial. This underlines the importance of consultation with scientists and clinicians independent of the researchers working on drug development, so that Phase I/IIa trial design is optimized to maximize the likelihood of success in clinical translation. We advocate global multidisciplinary stakeholder involvement as early as possible, in addition to traditional patient and public involvement, as a novel approach to drug development in stroke.

## Article Information

### Sources of Funding

This article was funded by National Institute for Health Research (Academic Clinical Fellowship 2022-26-004), Swiss National Science Foundation 310030_197823).

### Disclosures

Dr Galea and D. Bulters declare research funding and consulting fees from BioProducts Laboratory Limited and Evgen Pharma. Dr Galea received research funding related to haptoglobin from the Medical Research Council and Engineering and Physical Sciences Research Council. Drs Galea and Hugelshofer disclose consulting fees from CSL Behring. Drs Schaer and Hugelshofer received research funding from CSL Behring and are inventors on patents related to the use of haptoglobin.

### Supplemental Material

Literature search

Protocol

Figures S1–S3

## Supplementary Material



## Appendix

SAH Delphi Group collaborating authors: Amr Abdulazim; Andrew F. Alalade; Sheila A. Alexander; Sergi Amaro; Sepideh Amin-Hanjani; Christopher R. Andersen; Craig Anderson; Matthew H. Anstey; József Balla; Nourou Dine Adeniran Bankole; Judith Bellapart; Hemant Bhagat; Spiros L. Blackburn; Markus Brechmann; Paul W. Buehler; Jan-Karl Burkhardt; Yujie Chen; Jeremy Cohen; P. David Cooper; Liam G. Coulthard; Elisa Cuadrado-Godia; Joan Dalton; Anthony Delaney; Sylvain Doré; Jonathan Downer; Justin Dye; Isabel Fernandez-Perez; Oliver Flower; Béla Fülesdi; Ben Gaastra; Thomas Gaberel; James Galea; Gbetoho Fortuné Gankpe; Patrick Garland; Thomas Gentinetta; Magnus Gram; Jonas Heilskov Graversen; Patrick J. Grover; Daniel Guisado-Alonso; David Hasan; Adel Helmy; Julius Höhne; Isabel Charlotte Hostettler; Ajay Prasad Hrishi; Koji Iihara; David C. Irwin; Kiran Jangra; Aruma Jiménez-O’Shanahan; Richard F. Keep; Matthew Koch; Miikka Korja; Munish Kumar; Laura Llull; James J.M. Loan; Miguel Ángel Lopez-Gonzalez; R. Loch Macdonald; Shalvi Mahajan; Joan Martí-Fàbregas; Jose Medina-Suárez; Soren Moestrup; John More; Eghosa Morgan; Radhakrishnan Muthuchellappan; Paul Nyquist; Coralia Sosa Pérez; Promod Pillai; Nikolaus Plesnila; Jose Javier Provencio; Eamon Raith; Anna Ramos-Pachón; Scott B. Raymond; Luca Regli; Ynte Marije Ruigrok; Poonam Saharan; Edgar A. Samaniego; Gerrit Alexander Schubert; Ian Seppelt; Kamath Sriganesh; Jose I. Suarez; Jonathon Taylor; Nicole A. Terpolilli; Fernando D. Testai; Emanuela Tolosano; Ahmed K. Toma; Anderson Chun On Tsang; Andrew A. Udy; Florence Vallelian; Mariana Vargas-Caballero; Gregory M. Vercellotti; Mervyn D.I. Vergouwen; Michaela Waak; Hannah Warming; Peter C. Whitfield; George Kwok-chu Wong; Jason Wright; Adrian W. Zuercher.
