# Supplementary material for: Haptoglobin Treatment for Aneurysmal Subarachnoid Hemorrhage: Review and Expert Consensus on Clinical Translation
Source: Stroke. 2023 May 26;54(7):1930–42. doi: 10.1161/STROKEAHA.123.040205 (PMC10289236; doi:10.1161/STROKEAHA.123.040205)
Supplement: Supplementary file 1 [file str-54-1930-s001.pdf]

SUPPLEMENTAL MATERIAL for:

Haptoglobin treatment for aneurysmal subarachnoid haemorrhage: review and expert consensus on clinical translation

Ian Galea[1,2], Soham Bandyopadhyay[1,2], Diederik Bulters[1,2], Rok Humar[3], Michael Hugelshofer[4], Dominik J. Schaer[3]

for the SAH Delphi Group (see Appendix for author list)

1 Clinical Neurosciences, Clinical & Experimental Sciences, Faculty of Medicine, University of Southampton, Southampton, Hampshire, UK

2 Wessex Neurological Centre, University Hospital Southampton NHS Foundation Trust, Southampton, UK

3 Division of Internal Medicine, Universitätsspital and University of Zurich, Zurich, Switzerland

4 Department of Neurosurgery, Clinical Neuroscience Center, Universitätsspital and University of Zurich, Zurich, Switzerland

Literature search

Delphi Protocol

Figure S1

Figure S2

Figure S3

Literature search

We searched PubMed with the following keywords: (subarachnoid haemorrhage) AND (early brain injury OR delayed brain injury OR vasospasm OR delayed cerebral ischemia OR delayed ischaemic neurological deficit OR secondary brain injury OR outcome OR haemoglobin OR haptoglobin). Identified abstracts were screened by the authors. Further relevant references were identified from the bibliography of extracted articles.

Delphi study protocol

Aims

This collaboration was created to determine priorities for future research on aneurysmal subarachnoid haemorrhage (aSAH), specifically relating to extracellular haemoglobin (Hb) injury and potential treatment with haptoglobin (Hp). Using a modified Delphi process, this study primarily aims to ascertain if there is a consensus among experts in the field of aSAH that Hp should be pursued as a potential therapy. This study has the secondary aims of identifying the key research priorities for determining the role of Hb in aSAH, and ascertaining the safety and efficacy of Hp as a drug treatment.

The basic principles of the modified Delphi process include: (1) participants are professionals/experts in the field; (2) starting point is a questionnaire based on a literature review; (3) two or more rounds of feedback are performed during which participants can recalibrate their opinions based on overall feedback presented to them from previous rounds; (4) questionnaire revision between rounds reflects the developing consensus; (5) participant anonymity throughout.

Methods

Study Design and Participants

This is a Delphi study that will be conducted over two Rounds using an online Delphi manager software. A steering committee was formed to guide the process. Two stakeholder groups will be involved: 1) scientific experts in aSAH from academia and industry, and 2) clinician experts in aSAH (neurosurgeons, neurologists, and neurocritical care physicians).

Participants' inclusion criteria will be (1) experience with managing aSAH patients from the clinical disciplines of neurosurgery, neuro-intensive care and neurology, (2) scientific expertise (academic or pharmaceutical industry) in the field of haemoglobin/haptoglobin, or both. Participants will be asked to assign themselves to one of these groups as their primary expertise. Clinicians need to be actively involved in aSAH patient management, and will be asked to specify their clinical sub-specialty (e.g. neurosurgery, neurology, critical care, neuroradiology). Scientific experts need to have demonstrable evidence of working in the field of haemoglobin/haptoglobin within academia or the pharmaceutical industry as evidenced by their institutional affiliations, public profiles, and publications. Checks will be made by the steering committee to ensure alignment of this classification with publicly available websites and affiliations on publications and conference abstracts.

The two stakeholder groups will represent a wealth of experience in the field of aSAH and ensure views are captured from all aspects. Although there will be two stakeholder groups, they will co-participate in the same single Delphi process. Invited experts will be identified based on networks and track record in the field, using a network propagation technique whereby primary contacts will be encouraged to invite their contacts, and so forth, to minimise bias. An effort will be made to ensure global coverage.

Both Rounds will be conducted online. There will be two virtual meetings, before the first Round to explain the process and answer queries, and after the second Round to present the results. During the first Round, an online survey will ask participants from the two stakeholder groups to register with the online Delphi manager software and complete a series of questions. Two online meetings

will be held during Round One to facilitate. The questions will fall into two categories: a general category and a specific category, and participants will be asked to rank the importance of research themes in both categories. In the general category, they will also be asked to identify research priorities in the area, and examples will be provided in the specific category. Round Two will be another online Delphi survey, where participants who completed Round One will be asked to re-rank their answers after reviewing summary rankings and comments from all participants from the first survey, including any new themes emerging from the first Round.

All research themes will be ranked by participants regardless of the stakeholder group. All research themes included in Round One will be carried forward to the next Round regardless of the previous Round's rank. Each Round will be open for at least two weeks.

Sampling

There will be a centrally co-ordinated recruitment process. All elements of the recruitment process will be overseen, including study advertisements and recruitment letter distribution by email or in person. Participants will be invited to take part in both Rounds of the Delphi process from the beginning, but only participants who complete Round One will be eligible to participate in Round Two. The recruitment letter will fully explain the purpose of the research, but also the practicalities of the Delphi study and instructions on how to contact the research team for further information and the enrolment process. Importance of completing in full the Delphi process (all Rounds) will be stressed to avoid participant sample size attrition, and completion of the whole process will guarantee group authorship membership. Participation will be assumed if the patient logins into the Delphi software, registers and completes all tasks.

Sample Size

A pragmatic approach to participant sample size will be taken. There are no specific guidelines as to the optimal number of participants in a Delphi survey. Participation is required from both stakeholder groups in Rounds One and Two. Records of numbers recruited in each stakeholder group will be kept, and no new recruitment will occur once Round One of the Delphi process has ended.

Consent

Written consent will not be taken for completion of the questionnaire as part of the Delphi process. Consent will be implied through signing up to the online software. It will be made clear to the participant at the initial registration page that by submitting their details, they agree to participate in the Delphi process. Email addresses will be required as part of the Delphi survey but will be embedded into the software at the time of enrolment. Email addresses of participants will not be shared with anyone. Contact during Round Two via email will be a requirement of participation, and participants only have to sign up if they are happy to be contacted via email.

Questionnaires

The Delphi process will be run online using a Delphi Manager software. The questionnaire will be built into this software and sent out to all registered participants. On registering, participants will be asked to assign themselves to a stakeholder group and then some further data will be collected including their job roles. Participants will be asked about their beliefs regarding the need for a novel aSAH treatment, pathophysiological pathways determining secondary brain injury after aSAH and the role haemoglobin plays in these pathophysiological pathways, and research priorities in haptoglobin-based therapeutics. Participants will have to rank each of the research themes based on how important they see that research theme to be to them. Clear instructions will be provided on how to rank the research themes on the questionnaire and the time frames for doing so.

Scoring

Scoring will be via a ranking system, with research themes being graded by their level of importance.

Participants will only be presented with research themes that they should be able to rank. However, an open text box will be provided where participants can comment on whether they feel they did not have the expertise to rank a research theme appropriately.

Over the two Rounds of the Delphi process, the spread of ranks should reduce as consensus is reached. The definition of consensus for this project is outlined below. During the recruitment process, participants will be reminded of the importance of completing the whole Delphi. There will inevitably be some drop out from Round One to Round Two, and this will be recorded to calculate attrition rates between the Rounds.

Delphi Rounds

<u>Round One</u>

Participants from each of the stakeholder groups will be invited to rank all research themes. There will be an additional box for comments on why a rank has been given. At the end of the questionnaire, there will be an open-ended question asking participants whether they feel any further research themes, which have not been included, should be considered.

The total number of participants who participated in each stakeholder group will be recorded. For each research theme, the number of participants who felt they had the expertise to rank that research theme and the distribution of ranks will be summarised. The coordinator will review any additional comments, and these will be summarised and presented in Round Two. Any issues or conflicts arising from Round One will be addressed. This review will be done electronically. All research themes from Round One will be carried forward to Round Two and additional research themes recommended may also be included in Round Two.

<u>Round Two</u>

Participants who completed Round One in the time allocated will be asked to participate in Round Two. Each participant will be shown all the research themes and the feedback from Round One. Participants will be provided with the distribution of the ranks - with their rank highlighted - for each research theme. Descriptive comments for each research theme will also be presented and summarised. All feedback will be available to each participant, and it will be grouped by stakeholder

group. The participant will then be asked to re-rank each of the research themes based on the Round One feedback.

The total number of participants who participated in each stakeholder group will be recorded. For each research theme, the number of participants who felt they had the expertise to rank that research theme and the distribution of ranks will be summarised. The steering committee will review any additional comments, and these will be summarised. The ranks and comments for each research theme will be reviewed electronically.

Consensus Definition

The consensus definition has been decided before the Delphi study begins to avoid bias towards the beliefs of the research team running the study. The proposed definition of consensus for a research theme is described below:

- 'Consensus in' (a research priority): 70% or more participants ranking the research priority within the top 33% of their rankings AND fewer than 15% ranking it within the bottom 33% of their rankings.
- 'Consensus out' (not a research priority): 70% or more participants ranking the research priority within the bottom 33% of their rankings AND fewer than 15% ranking it within the top 33% of their rankings.
- ‘Consensus of equipoise’ (uncertainty about the importance of this research theme): Anything else.

For pathological pathways, consensus on contribution to secondary brain injury after SAH will be achieved if ≥70% of all participants rank the pathway within their top five ranks. For cell-free haemoglobin’s role in these pathways, consensus will be achieved if ≥70% of all participants score the pathway as “very important” or “extremely important”.

Post Delphi

The results of the Delphi process will be distributed to all invited participants. Research themes that have been categorised as 'consensus in' across the stakeholder groups during the Delphi will be included, and those outcomes categorised as 'consensus out' will be excluded. Research themes which have 'consensus in', 'consensus out', and 'consensus of equipoise’ discrepancies across the two stakeholder groups will be presented as such in the final text.

Dissemination

Following completion, the results of this study will be disseminated to the healthcare workforce and researchers who have a role in aSAH management. The results of this study will also be disseminated to non-government organisations with an interest in supporting patients with aSAH. Alongside this, the data will be presented at local, national and international conferences. This will facilitate the sharing of new ideas and practices that can be used to implement changes around the world. The final report will also be published. Individuals who complete both Rounds of the Delphi study will have group authorship status on the manuscript, and related conference presentations or other publications.

Figure S1. Drainage of haemoglobin-haptoglobin complexes via endogenous and exogenous pathways during intracranial treatment with haptoglobin via an external ventricular drain after aneurysmal subarachnoid haemorrhage. Endogenous drainage pathways include absorption through arachnoid granulations/villi into the venous system and drainage through the cribriform plate and via intramural peri-arterial drainage into the cervical lymph. Exogenous drainage pathways include via the external ventricular drain during unclamping, and via a lumbar drain.

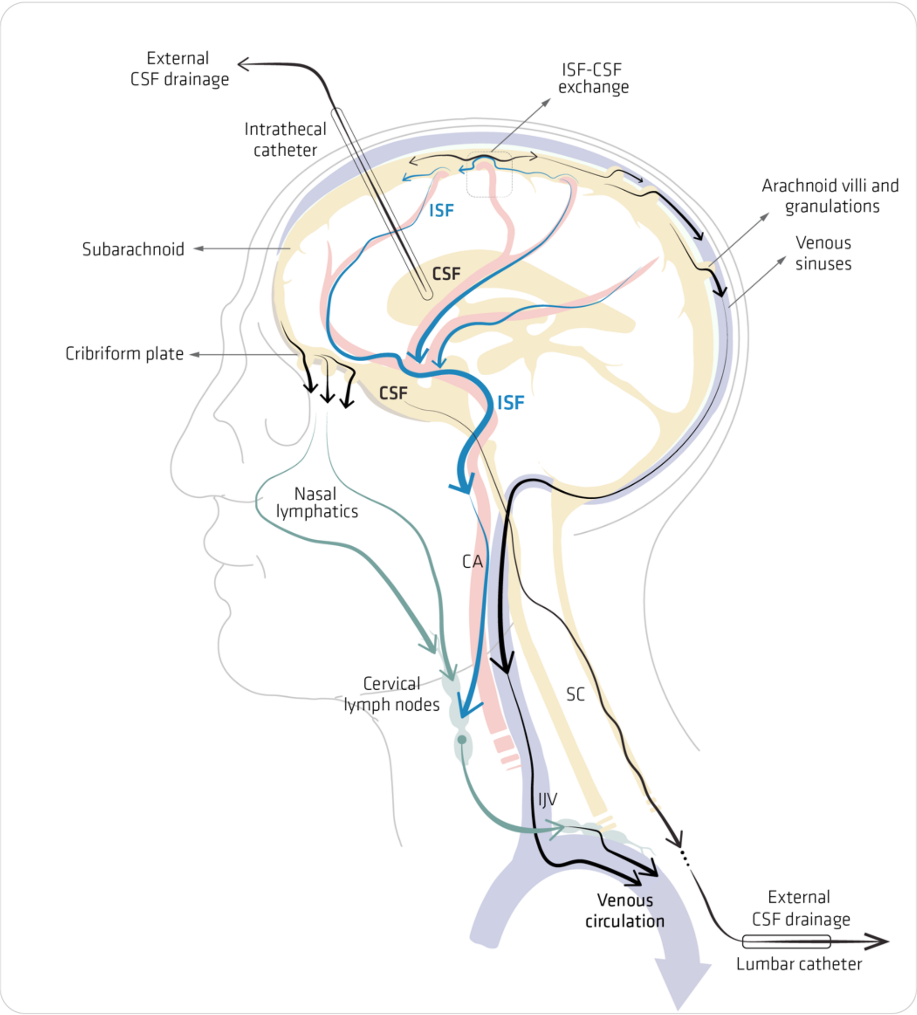

Figure S2. Clinicians

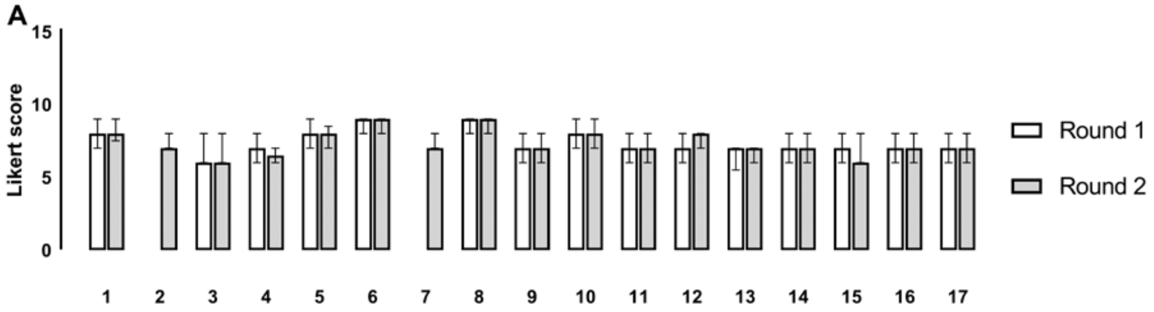

	1	2	3	4	5	6	7	8	9	10	11	12	13	14	15	16	17
Wilcoxon	*	NA	NS	NS	NS	*	NA			*	NS	*	NS	NS	NS	NS	NS
Mann-Whitney	NS	NA	NS	NS	NS	NS	NA			NS	NS	NS	NS	NS	NS	NS	NS

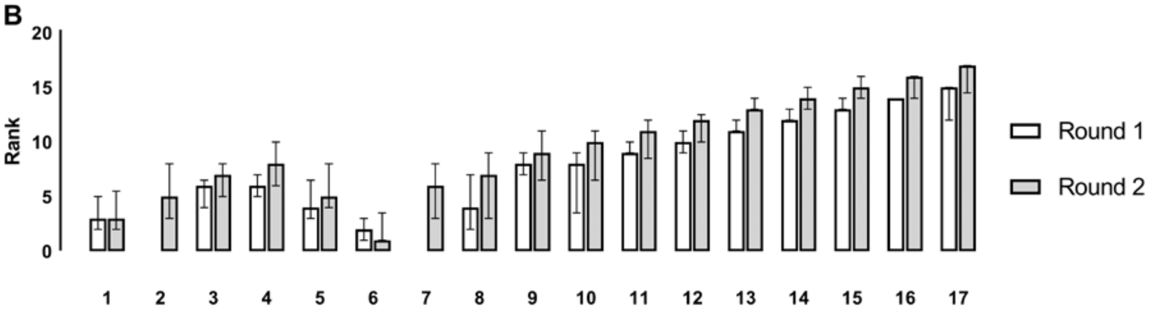

	1	2	3	4	5	6	7	8	9	10	11	12	13	14	15	16	17
Wilcoxon	NS	NA	****	****	***	NS	NA			****	**	****	****	***	****	***	****
Mann-Whitney	NS	NA	**	**	**	NS	NA			**	**	**	**	**	**	**	**

Research topics discussed for prioritization during haptoglobin translation development. Clinician participants were asked to **(A)** score the topics using a Likert system from 1 (extremely unimportant) to 9 (extremely important), and **(B)** rank the topics in order of priority. Research topics are numbered and a key is provided below. Data was analysed for change in individual responses (Wilcoxon) and change in group data (Mann-Whitney) between Delphi rounds. Data is shown as median ± interquartile range; * p<0.05, ** p<0.01, ** p<0.001, **** p<0.0001.

Key to topics discussed:

1. What is the optimal dose of intrathecal haptoglobin that should be administered following aneurysmal subarachnoid haemorrhage?
2. What is the dosing schedule for intrathecal haptoglobin?
3. Should the dose of haptoglobin administered intrathecally following aneurysmal subarachnoid haemorrhage vary according to the level of haemoglobin in the cerebrospinal fluid, as opposed to a fixed dose?
4. Can haemoglobin levels be estimated in the cerebrospinal fluid of a patient following aneurysmal subarachnoid haemorrhage in a non-invasive manner (e.g., through CSF sampling from an existing intrathecal device)?
5. When is the optimal time to administer intrathecal haptoglobin following aneurysmal subarachnoid haemorrhage
6. Is intrathecal haptoglobin safe to administer to patients following aneurysmal subarachnoid haemorrhage?
7. What are the characteristics of the target population who could benefit from treatment with intrathecal haptoglobin?

8. Which adverse outcomes of aneurysmal subarachnoid haemorrhage will be prevented or reduced following treatment with intrathecal haptoglobin?
9. Does the location of administration of haptoglobin impact its safety or efficacy?
10. Is haemoglobin responsible for secondary brain injury in patients with aneurysmal subarachnoid haemorrhage?
11. Is blood load correlated with poor functional outcome in patients with aneurysmal subarachnoid haemorrhage?
12. Would intrathecally administered haptoglobin be able to reach its site of therapeutic action in patients with aneurysmal subarachnoid haemorrhage?
13. Does the site of the blood clot affect the efficacy of haptoglobin administered intrathecally?
14. Does the presence of an external ventricular drain or a lumbar drain affect CSF circulation and/or pharmacokinetics of an intrathecally administered agent?
15. Is there an upper limit of CSF protein concentration that is safe?
16. Could increasing the CSF protein concentration precipitate hydrocephalus in a patient with aneurysmal subarachnoid haemorrhage?
17. Further insight into the exact mechanisms of injury from haemoglobin and/or haemoglobin/haptoglobin complexes before a Phase I trial

Figure S3. Scientists

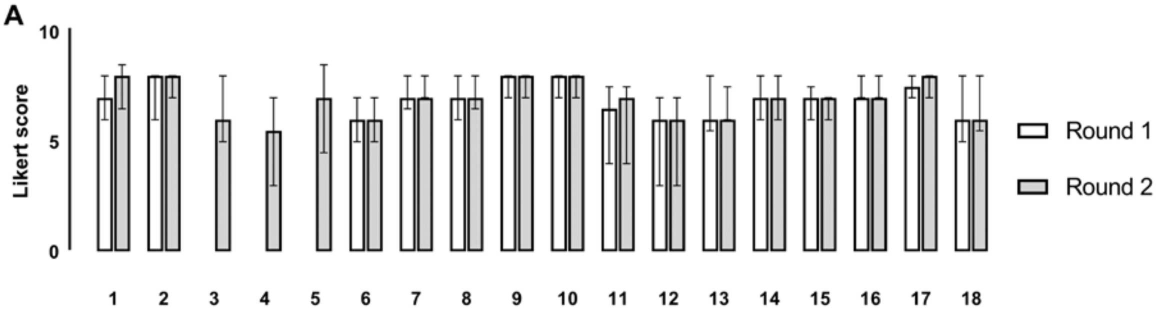

	1	2	3	4	5	6	7	8	9	10	11	12	13	14	15	16	17	18
Wilcoxon	NS	NS	NA	NA	NA	NS	NS	NS	NS	NS	NS	NS	NS	NS	NS	NS	*	NS
Mann-Whitney	NS	NS	NA	NA	NA	NS	NS	NS	NS	NS	NS	NS	NS	NS	NS	NS	NS	NS

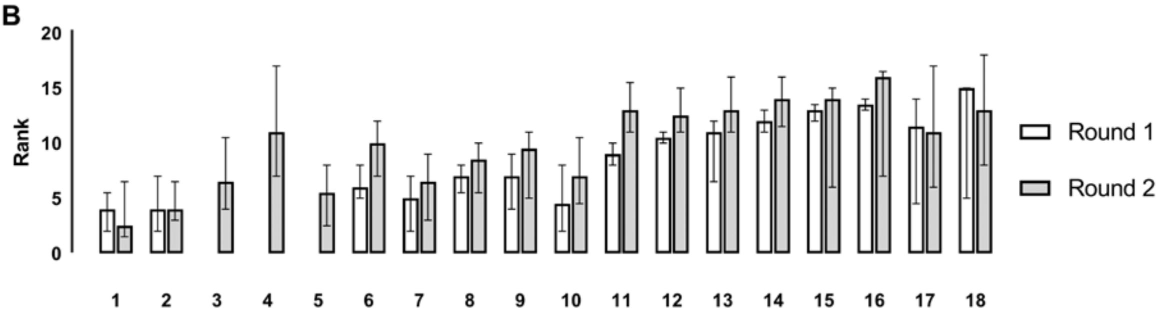

	1	2	3	4	5	6	7	8	9	10	11	12	13	14	15	16	17	18
Wilcoxon	NS	NS	NA	NA	NA	**	*	*	**	****	****	**	**	**	NS	NS	NS	NS
Mann-Whitney	NS	NS	NA	NA	NA	**	NS	NS	*	*	**	**	**	**	NS	NS	NS	NS

Research topics discussed for prioritization during haptoglobin translation development. Scientist participants were asked to **(A)** score the topics using a Likert system from 1 (extremely unimportant) to 9 (extremely important), and **(B)** rank the topics in order of priority. Research topics are numbered and a key is provided below. Data was analysed for change in individual responses (Wilcoxon) and change in group data (Mann-Whitney) between Delphi rounds. Data is shown as median $\pm$ interquartile range; * p<0.05, ** p<0.01, ** p<0.001, **** p<0.0001.

Key to topics discussed:

1. Should the dose of haptoglobin administered intrathecally following aneurysmal subarachnoid haemorrhage vary according to the level of haemoglobin in the cerebrospinal fluid, as opposed to a fixed dose?
2. Can haemoglobin levels be estimated in the cerebrospinal fluid of a patient following aneurysmal subarachnoid haemorrhage in a non-invasive manner (e.g., through CSF sampling from an existing intrathecal device)?
3. Can one use initial imaging to predict total haemoglobin exposure?
4. Should intrathecally delivered haptoglobin be combined with other therapeutic agents?
5. What are suitable surrogate radiological markers and/or biomarkers to assess the effectiveness of haptoglobin?
6. Does the phenotype of a therapeutic haptoglobin formulation affect the clearance of cell free haemoglobin after aneurysmal subarachnoid haemorrhage?
7. What is the impact of haptoglobin–haemoglobin complex formation on inflammation?
8. What is the impact of haptoglobin–haemoglobin complex formation on neuronal integrity?

9. What is the impact of haptoglobin–haemoglobin complex formation on iron deposition?
10. What is the pharmacokinetic model that best represents the distribution of haptoglobin following its administration?
11. What is the immunogenicity of haptoglobin?
12. Does the phenotype of a therapeutic haptoglobin formulation affect inflammation after aneurysmal subarachnoid haemorrhage?
13. Does the phenotype of a therapeutic haptoglobin formulation affect penetration into clot and brain tissue?
14. Are there sublethal effects of haemoglobin on neuronal transmission and plasticity?
15. Are the sublethal effects of haemoglobin reversible, naturally or with treatment?
16. Do the sublethal effects of haemoglobin contribute to functional outcome in patients with aneurysmal subarachnoid haemorrhage?
17. What is the clearance efficiency of haptoglobin–haemoglobin complexes in the cerebrospinal fluid following treatment with intrathecal haptoglobin?
18. Further insight into the exact mechanisms of injury from haemoglobin and/or haemoglobin/haptoglobin complexes before a Phase I trial
